# Supplementary material for: A new seasonal frozen soil water-thermal coupled migration model and its numerical simulation
Source: PLoS One. 2021 Nov 22;16(11):e0258861. doi: 10.1371/journal.pone.0258861 (PMC8608487; doi:10.1371/journal.pone.0258861)
Supplement: Attachment — Submitted filename: Response to Editor.docx [file pone.0258861.s005.docx]

We thank editors for reviewing this article, they put forward some very constructive opinions. We answer these questions one by one, the details are as follows:

Journal Requirements:

Please review your reference list to ensure that it is complete and correct. If you have cited papers that have been retracted, please include the rationale for doing so in the manuscript text, or remove these references and replace them with relevant current references. Any changes to the reference list should be mentioned in the rebuttal letter that accompanies your revised manuscript. If you need to cite a retracted article, indicate the article’s retracted status in the References list and also include a citation and full reference for the retraction notice.

**Answer:** Thank you very much for your advice. We re-searched the cited references and found that several of them were wrong, ref [2], [7], [17]. We have changed them, made corresponding references in the original text, and unified the format of all references. Please check the latest version of the manuscript.

Additional Editor Comments (if provided):
I have one comment on English and presentations. They should be improved significantly to meet the requirements for publication. For example, I read the Abstract of manuscript and feel that some expressions are not smooth or conventional or unclear.
**Answer:** Thank you very much for your advice.

After the last round of revision, the article has been greatly adjusted, mainly includes the sections of abstract, introduction and conclusion. The contents and words of these three parts are almost re-written, so we have translated the contents of these three parts again, after many modifications, the current version is formed.

The other sections of the article is that we hired American AJE company（https://www.aje.cn/)）, the translation and polishing have been translated by professional institutions, so there is little change.

Thank the editor again for carefully reviewing!
